# American Dental Association

**Published:** 1878-10

**Authors:** 


### AMERICAN JOURNAL

OF

### DENTAL SCIENCE.

**Vol. XII. THIRD SERIES—OCTOBER, 1878. No. 6.**

### ARTICLE I.

*The American Dental Association.*—Continued.

WEDNESDAY.—Evening Session.

Wednesday evening's session of the Dental Association opened at the appointed hour, and after the reading and approving of the minutes of the morning meeting, President Rehwinkle called for a report of the Committee on Credentials, and Dr. Shepard gave the names of delegates in attendance since the last report. He also presented several accounts, which were ordered paid.

The discussion of the papers presented on Histology and Microscopy were taken up, whereupon Dr. McQuillen took the floor and paid his respects to the voluntary contribution of Dr. Bodecker. He said he could not take it for granted that a man had made some wonderful discoveries unless the specimens were produced. "We want more original investigation and not so much of the text books," the speaker said: a man who makes a discovery, like a hen that has laid an egg, goes around cackling about it; and then somebody says here in such and such a book, there is a similar case. It is important that a familiar acquaintance with its literature should be had, in pursuing investigation upon a certain object.

Dr. Block spoke further of the manner of preparing specimens for the microscope. The speaker did not know that the mode recommended by Dr. Bœdecker was new, but he was not ready yet to take in all that was said with reference to the preparation of living matter. The Doctor spoke at some length with reference to the cells, but as his views were presented in a manner very scientific, they would not be of interest to the general public.

After further brief remarks by Dr. Dean, this order of business was passed and Dr. Fillebrown moved the adoption of the minority report of the Committee on Sections. Carried.

The discussion of the papers on Histology and Microscopy was again, on motion, declared in order.

Dr. Atkinson then addressed the convention and defended in an able and earnest manner what had been claimed in the paper of Dr. Bœdecker.

Dr. McQuillen followed with another emphatic appeal for original investigation, especially with the microscope, but begged that investigators would not start out with any preconceived theories.

This order of business being passed the report of the Committee on Pathology was called for and Dr. Atkinson read a strictly scientific paper, which was subsequently discussed by himself and Doctor Spalding.

The convention then adjourned until Thursday morning.

### THURSDAY.—Morning Session.

Pursuant to adjournment, the Convention was again convened yesterday morning, promptly at the appointed hour of ten o'clock.

Reports from the Committee on Dental Education was called for, and Dr. George A. Fredericks presented a report in which he held that the day of private tuition has gone by; that the public and the profession demand a collegiate education in dentistry. It being a specialty, dentistry should be taught by specialists in the art in actual practice, and a medical course ought to precede it. There was still room for improvement in the practical teaching in the infirmaries connected with colleges. The most important operation in dentistry was filling. Any one who set himself up to perform the operation and was not competent, was, in the estimation of the author, worse than a thief. "A thief only steals filthy lucre; while the dentist incompetent to fill a tooth properly adds abuse to injury, for he not only inflicts unnecessary pain but steals his patient's time and money with the assurance that the tooth operated upon is put in a condition for future preservation." Dr. Fredericks recommended that the President of the Association be authorized to appoint a committee of one at every place where a dental college is located to report to the Association the advantages possessed by such institutoin, for the practical teaching of the science and art of dentistry. A second report was next read by Dr. Thomas Fillebrown, in which he took the ground that the expediency and wisdom of medical schools including dentistry in their courses of instruction, appeared for the reason that more schools and those more widely distributed are needed, so that more inducements and facilities may be offered to young men to obtain a good education. When a professional school is located there will exist a higher standard of professional excellence. Where a dental school is established there will be superior operators and superior patients. In case a student makes a mistake in entering the practice of dentistry, being not well fitted by natural abilities for it, if medically educated he may correct his error by seeking some other branch of practice. And the contrary was also true. Many a poor doctor with surgical talent, but no opportunity to use it, might become a brilliant dentist. Dentistry as a specialty of medicine was perhaps more distinctly a branch of surgery, but in either case distinctly medical. When physi cians know as much about dentistry as they should, and when dentists were equally well informed with reference to medicine, all questions about recognition or mutual respect would cease to be raised, and "there would be no sickly sentimentality about claiming fellowship with a profession that disclaims us, for all will be of one family, owing the same *Alma Mater* and laboring for the same common good —the amelioration of human suffering and the elevation of the human race."

In concluding his report Dr. Fillebrown offered the following preamble and resolution :

Whereas, Dentistry is a specialty of medicine and requires, as do other specialties, a thorough medical education as preliminary to its most successful study and practice:

*Resolved*, That we deem it expedient and for the best interests of the practice of dentistry that existing medical schools so enlarge their curriculum as to include efficient instruction in dentistry, and thus make the requirements for graduation the same for this as for other specialties of medicine.

Dr. Crouse read a third report in which he urged that all the respectable colleges should form an Association and adopt certain regulations in regard to dental education and that the profession at large would endorse as high a standard as they might see fit to adopt. The State Examining Boards should not grant diplomas to those who are merely complying with the law. Dr. Crouse insisted that dentistry must be a specialty of medicine, and this can only be done by the students taking the degree of M. D. in the regular school of medicine. The dentist to fulfill his mission in the full sense of the term should be medically educated.

Discussion being in order Dr. Hunt said the subject was of great importance, but the objection to the proposition of Dr. Fillebrown, presented in his preamble and resolutions, was that it put the matter of education outside of the control of the dental profession. It was a mistake to suppose that medical instruction was not given in dental colleges. The success of a dental or medical college lay in the con scientious discharge by the officers of the duties and responsibilities resting upon them, and either institution was bad if not properly conducted. Whether dentistry was a distinct profession or not, was a subject that did not connect itself with dental education. Dr. Hunt gave notice that when the discussion was closed he should move the reference of Dr. Fillebrown's preamble and resolution to the section on education.

Dr. L. C. Ingersoll, of Iowa, said that in all professions the tendency had been downward instead of upward. In the effort to elevate the dental profession at this time, it seemed to him that the commencement was being made in the centre when it should be lower down at the beginning. It was elementary and not medical education which was wanting in dental education. There was too much made of the question of medical instruction and not enough of that pertaining to the elementary or starting point. There were young men who commenced the study of medicine who, at the same time, would be found incapable of writing three or four lines of good English. Go back to the old proverb, said the speaker, that "mind makes the man." All thought is in language. All that we know is in the medium of language. Insist on good mental acumen, or how will men be able to comprehend the terms in use.

Dr. Taylor, of Ohio, said that when he beheld the interest that was now demonstrated in dental education, it took his mind back to the time when education was denied to men. He advocated an extension of the present curriculum of study in the dental colleges. He believed that dental students should pass an examination in anatomy, physiology and pathology equal to that required of medical students. True dental science was true medical science.

Dr. J. Foster Flagg, of Philadelphia, said that Josh Billings, a well known and highly esteemed gentleman, had said "they are sensible people who think as we do," and he therefore esteemed the last speaker as a highly sensible gentleman. He favored a trial of the medical colleges.

The dental department of the Harvard University was the first inaugurated, and it was by all means the best; that of the University of Pennsylvania was the last, and it was decidedly the worst.

Dr. John Allen, of New York, one of the veterans of the profession, expressed himself as delighted with the advance which had been made in the matter of dental education since the days of his youth in the profession, when there was no such thing as dental schools and dental journals and conventions. A medical education was highly essential to the successful practice of dentistry.

Prof. Weatherbee, of Boston, desired to add his testimony to the general stock in trade regarding dental education, because it was someting which he had taken great pleasure in favoring. After a trial of dental colleges of forty years some men were desirous of putting the profession out to nurse, and recommended an education in medical colleges. They say it is necessary to success. It was a lie to say this —that was plain talk, but people could understand it. Students of dental colleges did not receive the honors of the institution until they were well up in a knowledge of the several branches which they would pursue in a medical college. Students should first graduate in a university; then study letters, theology, and the law, before they pay attention to dentistry. Following this they should take a three years course of medicine.

Dr. Darbee, of Philadelphia, a representative of the University of Pennsylvania, waiving anything that had been said of the institution that was not complimentary to it, said for the encouragement of those who recommended a medical education, he would state that a majority of the students at the University had signified their intention to take the three years course in medicine.

Time, he thought, would prove whether the medical education was necessary. The course recommended by Dr. Fillebrown and Dr. Weatherbee would make a man taking them, forty or fifty years old, before he would be able to do anything; before he had lived more than half his life, he would be ready for his grave. Dr. Darbee thought a young man should become a graduate in medicine, and then he could practice dentistry if he wished.

Dr. Atkinson said dentistry was medicine, and he desired to put this forth as a dogmatic aphorism.

Dr. Spaulding of the Western College of Dental Surgeons, said there was a uniformity of opinion as to whether dentistry was medicine, and whether a medical education was necessary for a dental education. The question was how far the rule could be applied and enforced. The average with dental graduates to-day was better than with medical graduates. The proposition of Dr. Fillebrown would result in the dental profession being swallowed up in medicine, and it would cease to be a distinct profession. It was old enough to walk alone, at this time, and then was named of taking steps which would fuse it into the medical profession. There was need of unanimity on the subject, or the results would be very disastrous.

Dr. McQuillen favored a medical education—the broadest education for a dentist; but the question was how far it was practicable at this time. The recognition accorded to American dentistry was due to the men in and out of the dental colleges. If universities desired to inaugurate dental departments they should demand the full course of three years.

Dr. Ingersoll again took the floor, and said dentistry is a specialty of medicine, but the dental profession asked no favors of the medical profession, and had been offered none.

After further remarks from one or two gentlemen, Prof. Buckingham moved the previous question.

The question being taken on the motion to adopt the preamble and resolution of Dr. Fillebrown, the same was lost by a vote of 28 to 19.

The discussion was then continued, and Dr. Crouse made a lively attack on what he termed one-horse dental schools, and said no school should be endorsed which did not provide the full course.

The subject of dental education was now passed.

The Association then adjourned for the extra session.

THE EXTRA SESSION

opened at four o'clock with Dr. Rehwinkle in the chair. Dr. Odell, of New York, was made Secretary *pro tem.*, but soon surrendered the office, upon the arrival of the regular incumbent, Dr. Dean.

Dr. Weatherhill moved that the certificate of membership reported by the Corresponding Secretary, be adopted.

Dr. Sheppard moved as an amendment that the certificate be adopted provided that the word Honorary was prefixed to that of Member.

Some animated discussion ensued, in which it was held that the issuing of a certificate of membership to permanent members would result in some unprincipled person making improper use of it; also that men granted the certificate would in time fall in arrears, and finally never put in an appearance; but that certificate would be found framed and hung up in their office. The prudence and judgment demanded that the amendment should prevail seemed to be the prevailing sentiment, although the original motion was ably and earnestly defended by the mover, and several others.

Dr. Rehwinkle called Dr. Sheppard to the chair, and moved as an amendment to strike out the words "honorary members," and add the words, "provided that permanent members who have been regular members for five years, and are in good standing, shall, on application to the Secretary and Treasurer of this society, be entitled to the certificate of membership."

Dr. Spaulding offered the following as a substitute for the original motion and the amendment to the amendment:

*Resolved*, That the certificate of membership of this Association shall be conferred on honorary members.

The resolution was declared out of order.

Dr. Rehwinkle withdrew his former amendment and offered the following as a substitute for the whole question :

*Resolved*, That this certificate be granted to honorary members, and to all worthy members of this Association, provided they have retained their membership for five consecutive years, upon the payment of $—, the cost of the certificate.

After some further debate the previous question was ordered, and a vote was taken on the substitute of Dr. Rehwinkle, which was carried by a vote of 18 to 19.

It was then moved by Dr. Crouse to lay the whole matter on the table. Carried. Ayes 30, noes 17.

It was moved that the engraved plate be placed in the possession of the Secretary. Carried. The bill for the engraving of the plate was ordered paid.

Dr. Rehwinkle having resumed his position as presiding officer, Dr. Spaulding offered the following resolution and moved its adoption :

*Resolved*, That a committee of five on revision and engrossment of the constitution be appointed by the Chair. In case no new amendments to the constitution are proposed by said Committee they may at their discretion cause to be printed five hundred copies of the constitution. This Committee shall report at the earliest practicable time.

Dr. Crouse moved that the matter be referred to the publication committee, with power to act. Lost.

The resolution was then adopted.

Dr. Taft moved that a committee be appointed to draft an appropriate memorial to the late Dr. George T. Barker, who was the second Vice-president of the association.

Dr. Rawls moved as an amendment to go into a committee of the whole on the subject.

Dr. Crouse moved to lay the amendment on the table; it was withdrawn by Dr. Rawls.

The original motion was then put and carried.

The chair announced as the committee, Dr. Taft, of Cincinnati, Dr. Cushing, of Chicago and Dr. Gorgas, of Baltimore.

On motion of Dr. Shepard the Publication Committee were instructed to incorporate the constitution in the transactions of the Association.

On motion of Dr. Taft, Prof. Buckingham was added to the Memorial Committee.

The Association then adjourned until 7.30 P. M.

THURSDAY.—EVENING SESSION.

Thursday evening's session opened at eight o'clock, Dr. Rehwinkle in the chair.

The President announced that a telegram had been received from Dr. Dennis, of California, asking that the selection of the next place of meeting be postponed until his arrival.

Dr. Taft therefore moved that the selection of the next place of meeting be deferred until Friday morning at ten o'clock.

Dr. Fillebrown moved to lay the motion on the table. Carried.

Dr. Rawls moved a reconsideration of the vote last taken. Lost.

The Nominating Committee reported that Niagara Falls was the only place that had been named for the holding of the next Convention.

Cincinnati, Saratoga, Watkins, Manhattan Beach, Boston, Atlanta and Long Branch were also named by members of the Association.

On motion the nominations were closed and the Association proceeded to ballot, Doctors Rawls and Black being appointed as tellers.

On motion it was ordered that after the first ballot all places except the three receiving the highest number of votes be dropped.

On the first ballot the contest was narrowed down to Niagara Falls, Cincinnati and Saratoga, the two first named having an even number of votes.

On the second ballot out of seventy-five votes, Niagara Falls received thirty-seven, Cincinnati twenty-six and Saratoga twelve. Saratoga was dropped.

On the third ballot Niagara Falls came out victorious by fifty-eight votes out of seventy-seven.

The election of officers was next in order, and the members of the Association made choice as follows:

*President.*—Dr. H. McKellops, St. Louis.

*First Vice-President.*—Dr. L. D. Shepard, Boston.

*Second Vice-President.*—Dr. L. Dr. Carpenter, Atlanta, Georgia.

*Corresponding Secretary.*—Dr. A. O. Rawls, Kentucky.

*Recording Secretary.*—Dr. George A. Cushing, Chicago.

*Treasurer.*—Dr. W. H. Goddard, Louisville.

*Executive Committee.*—Drs. C. N. Pierce, G. V. Black, J. W. Keeley.

With the close of the election the Association adjourned.

FRIDAY.—Morning Session.

The Association opened at ten o'clock, A. M.

The report of the Committee on Therapeutics being called for, a paper was read by Dr. F. M. Odell, and discussed by Doctors Spalding, Flagg and Atkinson.

Operative Dentistry was the next subject iaken up, and a lengthy paper was read by Dr. M. H. Webb, and referred to the Committee on Publication.

A second paper on the same subject, was read by Dr. D. D. Smith and referred.

Dr. Shepard was called to the chair.

Dr. J. Foster Flagg then read a paper on "Plastic Filling, as a Power in Dentistry," in which he stated that for nearly two years he had not employed one sheet of gold foil, and had completely abandoned its use. He insisted that for certain kinds of defined teeth and certain kinds of defined cavities, where it is claimed gold is the best filling, there are other kinds of filling, of a plastic character, that are better than gold.

Dr. McQuillen directed attention to two specimens prepared by Dr. Holloway, of La Porte. He said in preparation of specimens the means used in mounting them too often destroy the normal structure and have often led to the most erroneous interpretations.

Dr. Fredericks followed with a paper in which he discussed the asserted necessity of keeping the cavity of a tooth dry during the operation of filling. He cited cases in which he had successfully plugged a tooth with gold under saliva.

Dr. Stockton read an additonal paper under the head of operative dentists, in which he recounted the wisdom drawn from numerous failures of a varied character. At its conclusion, the hour of adjournment having arrived a recess was taken until half-past three o'clock.

### FRIDAY.—Afternoon Session.

The Association re-assembled at the appointed hour.

The subject of a certificate of membership being taken from the table, Dr. Crouse moved that the design of the certificate submitted be accepted for the use of honorary members, and the words "honorary member" be engraved in the plate.

Dr. Barrett criticized the tabling of the subject on Thursday as unfair and said that he thought it unjust, with the slim attendance at this time, to take advantage of the absence of those opposed to the matter, for the purpose of pushing it through.

Dr. Crouse moved that the discussions in relation to the certificate on Thursday be stricken from the minutes. Dr. Barrett opposed the motion. The gentleman making the motion had foolishly wasted the valuable time of the convention in the discussion, and his remarks ought to go on record.

Dr. McQuillen added a word in favor of the motion.

The Secretary gave notice that there was no record of the remarks of the gentlemen who participated in the discussion.

Dr. Crouse thereupon withdrew his motion.

On motion it was resolved that a seal for the use of this Association be procured.

The installation of the officers elect, was next in order, and was carried out in due form.

In vacating his chair, the President, Dr. Rehwinkle, gracefully presented his acknowledgments for the support accorded him by the members of the Association, and expressed his best wishes for the future welfare and prosperity of the Association. He then introduced the newly.elected President, Dr. McKallops, who, upon assuming his position, returned his thanks for the confidence manifested in his ability by his election to the Presidency. He deemed it a high honor, and appreciating the responsibilities of the position, hoped to be able, with the assistance of those who had placed him in the chair, to satisfactorily and properly discharge the duties devolving upon him.

Returning to the regular order of business, the report of the Special Committee on Chemistry was called for.

Dr. Cushing read a report from Dr. Moel, of San Francisco, California. It treated of the destructive character of sugar acids on the teeth, and presented suggestions as to the conclusions to be drawn therefrom. It also discussed and took exceptions to the assertion that sulphuric and nitric acid is generated by the putrification of meats between the teeth, and in conclusion took the ground that the greater part of dental decay arises from the decomposition of starchy and sacharine substances in the mouth.

The subject of chemistry being passed—

Dr. C. D. Cook read a report on dental literature of recent issue, and criticized the various publications, both foreign and domestic, giving the important questions upon which they treat, and their relation to the profession.

Discussion being in order, remarks were made by Doctors McQuillen and Atkinson.

Dr. Shepard moved a suspension of business. Carried.

The various sections were duly organized as follows:

*Section* 1. Artificial Dentistry, Metallurgy and Chemistry—Chairman, Dr. Finley Hunt, Washington, D. C.; Secretary, Dr. C. S. Stockton, Newark, N. J.

*Section* 2. Dental Education—Chairman, Dr. J. N. Crouse, Chicago.

*Section* 3. Dental Literature and Nomenclature—Chairman, Dr. W. H. Atkinson, New York; Secretary. Dr. J. Taft, Cincinnati.

*Section* 4. Operative Dentistry—Chairman, Dr. I. J. Weatherbee, Boston; Secretary, Dr. W. H. Goddard, Louisville, Ky.

*Section* 5. Anatomy, Physiology, Histology, Microscopy and Etiology—Chairman, Dr. M. S. Dean, Chicago; Secretary, Dr. M. H. Webb, Lancaster, Pa.

*Section* 6. Pathology, Therapeutics and Materia Medica. Chairman, Dr. F. H. Rehwinkle, Chillicothe, O.; Secretary, Dr. Will Taft, Cincinnati.

The reception of the report of the Committee on Credentials was received and accepted.

The Committee on Prize Essays reported that they had received one essay only, which, they had been informed by the author, was the same one before the Committee last year and rejected by them. The Committee therefore did not feel warranted in reversing their decision.

The report was accepted and adopted.

The Committee on Appliances made a report of the new and useful inventions and appliances of the past year for the lightening of the labors of the operator and to aid him in performing his work.

It was ordered that the report take its usual course.

The special committee to whom was referred the amendment to the constitution relative to dental colleges reported that they did not deem it expedient to make any such change in the constitution. They offered the following resolution for adoption:

*Resolved*, That this Association disapprove of the practice of dental colleges in graduating upon less than two full courses of lectures, and believe it to be detrimental to the interests of dental education, and advise the omission of such inducements from their annual announcements.

Dr. Shepard moved a reconsideration of the action taken in adopting the resolution of Dr. Spalding providing for the appointment of a Committee on the printing of the by-laws. The resolution was then revised so as to read "that a committee of five on revision and engrossment of the constitution be appointed by the Chair. This Committee shall report at the earliest practicable time." The resolution as amended was adopted.

The committee appointed to draft resolutions expressive of the sense of the Association, on the death of the late Dr. George T. Barker, presented the following, which was adopted:

WHEREAS, In the wisdom of Divine Providence, our colleague, Professor George T. Barker, Vice-President of this Association, has been removed by death from our midst, in the prime of his manhood; therefore

*Resolved*, That we feel called upon to testify, in this manner, our appreciation of his usefulness as a member of our profession.

*Resolved*, That we deplore his loss and regard his example, both professional and social, as eminently worthy of emulation.

*Resolved*, That the sympathy of the members of this Association be and hereby is tendered to the bereaved family of our lamented brother.

*Resolved*, That the above testimonial be inserted in the minutes of this Association and printed on a memorial page in the annual transactions.

J. TAFT,
F. J. S. GORGAS,
GEORGE H. CUSHING,
T. L. BUCKINGHAM,
*Committee.*

A vote of thanks was tendered to the retiring officers of the Association, to *The Buffalo Courier*, for its extended and complete reports of the sessions of the convention, and to which we are indebted for the substance of this report; and thanks were voted to the hotels, the railroads and all others for courtesies extended to members of the Association.

The recommendation of Dr. Weatherbee, during the discussion on dental education, on Thursday, that students study law, theology, etc, should be accepted as being made in an ironical sense. This explanation is made in view of the fact that a number thought he was really in earnest in what he said.

The minutes of the session were read and approved and the Association then adjourned until 1879.

This Session of the American Dental Association, was one of the most pleasant we have ever attended, and the large number of dentists in attendance, in addition to the regular delegates, added much to the pleasure and interest of the occasion.

